# Designing the Next Generation of Vaccines: Relevance for Future Pandemics

**DOI:** 10.1128/mBio.02616-20

**Published:** 2020-12-22

**Authors:** Jorge Domínguez-Andrés, Reinout van Crevel, Maziar Divangahi, Mihai G. Netea

**Affiliations:** a Department of Internal Medicine, Radboud University Nijmegen Medical Centre, Nijmegen, The Netherlands; b Radboud Center for Infectious diseases (RCI), Radboud University Nijmegen Medical Centre, Nijmegen, The Netherlands; c Radboud Institute for Molecular Life Sciences, Radboud University Medical Center, Nijmegen, The Netherlands; d Meakins-Christie Laboratories, McGill University Health Centre, Montreal, Quebec, Canada; e Department of Medicine, McGill University Health Centre, Montreal, Quebec, Canada; f Department of Microbiology and Immunology, McGill University Health Centre, Montreal, Quebec, Canada; g Department of Pathology, McGill University Health Centre, Montreal, Quebec, Canada; h McGill International TB Centre, McGill University Health Centre, Montreal, Quebec, Canada; i Department for Genomics & Immunoregulation, Life and Medical Sciences Institute (LIMES), University of Bonn, Bonn, Germany; Albert Einstein College of Medicine

**Keywords:** vaccination, metabolism, epigenetics, modulation, trained immunity, amplifier

## Abstract

The development of vaccines is one of the greatest medical interventions in the history of global infectious diseases and has contributed to the annual saving of at least 2 to 3 million lives worldwide. However, many diseases are not preventable through currently available vaccines, and the potential of modulating the immune response during vaccination has not been fully exploited. The first golden age of vaccines was based on the germ theory and the use of live, attenuated, inactivated pathogens or toxins. New strategies and formulations (e.g., adjuvants) with an immunomodulatory capacity to enhance the protective qualities and duration of vaccines have been incompletely exploited. These strategies can prevent disease and improve protection against infectious diseases, modulate the course of some noncommunicable diseases, and increase the immune responses of patients at a high risk of infection, such as the elderly or immunocompromised patients. In this minireview, we focus on how metabolic and epigenetic modulators can amplify and enhance the function of immunity in a given vaccine. We propose the term “amplifier” for such additives, and we pose that future vaccines will have three components: antigen, adjuvant, and amplifier.

## INTRODUCTION

In the last 100 years we have witnessed how local disease outbreaks of different natures can quickly transform into global threats as they spread through the world. The agents of Spanish flu, HIV, Dengue, Zika, Ebola, Middle East respiratory syndrome (MERS), severe acute respiratory syndrome coronavirus (SARS-CoV), or SARS-CoV-2 have taken tens of millions of lives in the last century (1). In an increasingly globalized and densely populated world, the consequences of the spread of new diseases can have an unprecedented impact on humanity in the next decades. Therefore, it is crucial to count on novel powerful tools to stop the transmission and reduce the incidence of future pandemics. In this regard, the most powerful allies to impede the spread of diseases are vaccines. From variolation experiments in ancient India and China to the experiments of Jenner and Pasteur until the most modern vaccines against cancer, the discovery of vaccines, the development of vaccination strategies, and global immunization programs have been exceptional worldwide breakthroughs with a large impact on public health. The discovery of the benefits of vaccination was prior to the rise of immunology as a new field of discovery at the end of the 19th century, when Ehrlich and Metchnikoff, respectively, discovered antibodies and described the mechanisms of phagocytosis, paving the way for the further development of humoral and cellular immunology (2). Since then, the progress of vaccination has been parallel to the growth of fundamental and translational immunology. Vaccines are among the public health measures with the greatest benefits to humanity, preventing major epidemics, deaths, and sequelae. Additionally, mass vaccination generates herd immunity, so diverse groups beyond vaccinated people benefit from it, including immunocompromised individuals (e.g., elderly or pregnant women) that cannot receive a vaccine due to the risk of developing infections.

However, the development of effective vaccines to combat other widespread infections, including tuberculosis (TB), HIV, and malaria, lags behind the enormous economic and political efforts invested. While this is beyond the scope of the current minireview, the growth of antivaccination movements is alarming, contributing to the reemergence of diseases that were well controlled. One such example is measles, which has shown a 30% increased incidence in recent years, including in regions where it was considered extinct (3). Undoubtedly, our current approach for vaccination requires new strategies and formulations that take advantage of the immunomodulatory actions of the different ingredients of vaccines to unleash the full potential of the immune system. Fine-tuning the immune-mediated effects of vaccines may be employed not only to improve the responses against infectious diseases but also to prevent or modulate the course of some noncommunicable diseases (NCDs), such as diabetes, cancer, or inflammatory and autoimmune diseases.

While the concept of vaccines is often linked to adaptive immune responses in vertebrates, innate immunity in simple organisms, such as plants and invertebrates, as well as in complex organisms shows memory-like capacity, termed trained immunity (4). The molecular mechanisms involved in the generation of trained immunity are mediated via stable and durable epigenetic and metabolic changes, leading to enhanced immune responses against the same or different pathogens (5). These nonspecific responses can enhance the responsiveness of the immune system against a variety of pathogens, so they can be an excellent tool to prevent the transmission and reduce the incidence of future diseases for which a specific vaccine is not available.

In recent years, we have witnessed a rapid development of cutting-edge techniques, such as metabolomics and genetic analysis, that have widely increased our understanding of the mechanisms involved in the immune-mediated protection produced by vaccines. The metabolic and epigenetic reprogramming of both innate and adaptive immune responses has become the cornerstone for developing vaccines (6, 7). Targeting metabolic and epigenetic mechanisms offers an excellent opportunity to develop a new generation of vaccines with improved efficacy and safety. In this minireview, we discuss the potential of these novel approaches for the development of vaccines against emerging multiresistant pathogens, as well as NCDs.

## AN EFFECTIVE VACCINE REQUIRES BOTH INNATE AND ADAPTIVE IMMUNITY

The concept of vaccination is classically associated with adaptive immune responses, as B cell and antibody responses are the cornerstone of a vast majority of the current effective vaccines in humans (8). The relative immunogenicities of vaccines vary from vaccine to vaccine, depending on the adjuvant and type of vaccine used: live attenuated, inactivated, subunit, or toxoid vaccines. The vaccination process triggers a complex interaction between antigen-presenting cells (APCs) and naive T and B cells (9). Following vaccination, activated APCs migrate to draining lymph nodes, activating T and B cells. Activated B cells proliferate and undergo somatic hypermutation and isotype changes in the immunoglobulins on their surface, making it possible to select the B cells that recognize the vaccine antigens with the greatest affinity (10). Once the B cells are activated, they evolve into antibody-producing plasma cells or into long-lasting memory B cells expressing high-affinity receptors for a specific vaccine antigen on their surface. As with memory B cells, some activated T cells give rise to memory T cells, which trigger powerful immune responses after reexposure to the same antigen (11).

**FIG 1:**
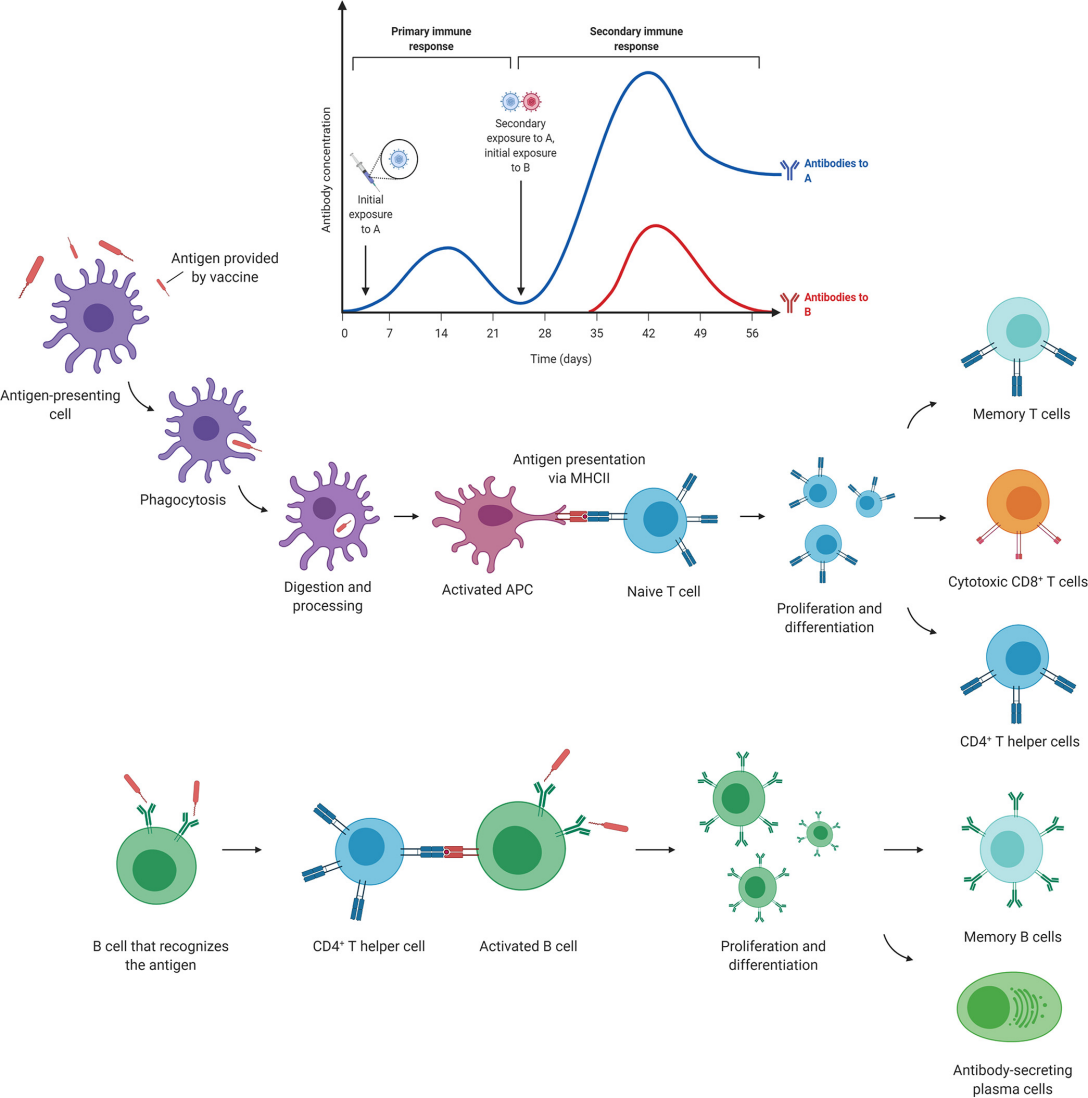
Mechanisms of classical vaccines. Inactivated pathogens contained in classical vaccines are phagocytosed by antigen-presenting cells (APC) and presented to naive T cells in the lymph nodes. Antigen presentation triggers the activation and proliferation of T cells and their differentiation into different subsets, namely, CD4^+^ helper T cells, CD8^+^ cytotoxic T cells, and a group of memory T cells, which will persist in the circulation and the tissues for a long time and will confer immunological memory after exposure to the same pathogen. For their part, B cells get activated after direct recognition of the pathogen and contact with a CD4^+^ helper T cell that was previously activated by an APC. This specific B cell will undergo clonal expansion and give rise to plasma cells, which produce large amounts of specific antibodies against the antigen contained in the vaccine, and long-lasting memory B cells.

However, the activation of the adaptive immune system relies on the instruction of the innate immune system (12). Innate immune cells express pattern recognition receptors (PRRs) that bind to evolutionarily preserved structures of pathogens (pathogen-associated molecular patterns, or PAMPs). This early recognition by innate cells activates a series of intracellular signaling pathways that lead to the initiation of immune responses, including phagocytosis, the production of reactive oxygen species (ROS), and the secretion of immunomodulatory chemokines and cytokines (13). Antigens are presented to lymphocytes through the major histocompatibility complex (MHC), contributing to the formation of an immune synapse with the T and B cells, triggering the activation of the adaptive immune responses (14) (Fig. 1).

Live attenuated vaccines are generally the most immunogenic formulations (15). They show many similarities with their pathogenic counterpart, spreading through the organism, reaching their target tissues, and stimulating the immune cells in a natural manner. Oral and nasal vaccines are able to replicate in mucosae in the first hours after vaccination, mimicking natural infection (16). Live attenuated vaccines also express on their surface numerous PAMPs that profoundly activate innate immune cells, generating local and systemic immune responses. Non-live vaccines are based on inactivated microbes, proteins, or microbial structures that act as antigens. The immunogenicity of recombinant-protein-based vaccines is very low, and therefore, non-live vaccines often require repeated vaccination or the presence of an adjuvant to increase the initial stimulation and activation of APCs at the site of vaccination (17). Thus, the development of a new generation of adjuvants and immune modulators can enhance the immunogenicity of non-live vaccines to increase the efficacy of vaccination and reduce the number of doses necessary to afford full immunization.

Importantly, live attenuated vaccines trigger strong, systemic responses that generate long-term effects, not only in adaptive immunity but also in the innate immune system through the induction of trained immunity (6). For instance, it has been shown that measles, polio, or bacillus Calmette-Guérin (BCG) vaccinations are able to induce long-term metabolic and epigenetic reprogramming of the innate immune cells, granting protection against both homologous and heterologous infections (5). In line with this, BCG vaccination has been repeatedly shown to reduce overall mortality in children from countries with high infectious burdens (>50 to 60%), through reduction of respiratory tract infections and neonatal sepsis (18). Similarly, measles vaccination increases the production of proinflammatory cytokines 1 month after vaccination (19), and it is linked to a 30 to 86% reduction in the overall risk of death in children from low-income countries (20). The effects of measles vaccination are likely not only due to trained immunity but also a consequence of the protection from the long-term immunosuppression caused by the measles virus (21). Altogether, the measles vaccine protects the host against pathogens different than the measles virus, through increasing the inflammatory responses and preventing the immunosuppression caused by the measles infection. These effects are not restricted to Africa, since a group of Danish children who received the live measles-mumps-rubella (MMR) vaccine showed lower hospitalization rates due to any infection than children who had received the inactivated diphtheria, tetanus, pertussis-inactivated polio virus-Haemophilus influenzae type b (DTaP-IPV-Hib) vaccine (22). These broad, protective effects of live attenuated vaccines can be used to decrease the impact of diseases for which a specific treatment is not available. For example, intravesical instillations with BCG are a standard treatment for early-stage bladder cancer (23), while a recent study showed that elderly individuals vaccinated with BCG present a lower rate of new infections, especially in the respiratory tract, than unvaccinated individuals (24). In this regard, there are multiple ongoing clinical trials to assess the potential of BCG to reduce the impact of CoV disease 2019 (COVID-19) (25). The induction of trained immunity can be employed to attenuate the impact of diseases without an effective treatment and be used as a bridge to reduce their incidence and transmission until a specific vaccine is available.

## EPIGENETIC AND METABOLIC CHANGES IN RESPONSE TO VACCINATION

Mounting an efficient immune response requires the adaptation of the immune system at different levels. Upon infection or vaccination, the immune cells initiate a cascade of intracellular events that lead to antigen presentation, cell proliferation and differentiation, and the production of different soluble factors, such as cytokines and chemokines, to maintain or amplify the response (26). Thus, the regulatory mechanisms involved in immune metabolism become central for maintaining the cellular demand under these conditions (4). Circulating and lymph node B and T cells are in a quiescent state until they are stimulated by activated APCs. During steady state, they present low biosynthetic demands, with minor metabolic demands, relying on the oxidation of glucose through oxidative phosphorylation and fatty acid oxidation for generating energy (27, 28). These metabolic pathways are relatively slow but very efficient, extracting a large amount of energy from glucose and fatty acids via mitochondrial electron transport chain. However, when lymphocytes are activated, they quickly need to proliferate, produce, and release various proteins (antibodies in the case of B cells, cytokines in the case of T cells) or induce cytotoxic responses (29). These functions require the instant availability of large quantities of ATP and the availability of lipids for membranes and nucleic acids, as well as induction of protein synthesis. Subsequently, immune cells, such as lymphocytes, rewire their metabolism toward glycolysis, which provides them with a fast supply of energy. They also increase the activities of other metabolic pathways, including protein synthesis, inositol phosphate metabolism, glycerophospholipid metabolism, and sterol metabolism (30), which can act as alternative sources of energy and matter. Cells from the innate immune system undergo similar processes. Neutrophils are short-lived cells whose main function is to phagocytose and kill pathogens, so they present a low number of mitochondria with a highly glycolytic metabolism (31). On the other hand, monocytes, dendritic cells (DCs), NK cells, and macrophages rely mainly on oxidative phosphorylation coupled to the electron transport chain while they are resting or “patrolling” (32, 33). As soon as the pathogens or vaccines are sensed by their PRRs, these cells experience an increase in the activities of diverse metabolic pathways, such as glycolysis and glutaminolysis, to fulfill their high metabolic demands upon activation (34). The induction of long-term responses to vaccination depends on epigenetic remodeling in monocytes/macrophages and NK cells, whose genome keeps the open conformation of the promoters and/or enhancers of proinflammatory genes, which facilitates an enhanced responsiveness after restimulation with the same or a different stimulus (6). These changes can be maintained in time due to stable and durable epigenetic modifications of cells from the hematopoietic progenitor niche in the bone marrow, which transmit their changes to the various immune cell populations, allowing the maintenance of enhanced responses for months or even years (35).

The metabolic adaptation of cells after vaccination is paralleled by the modification of the epigenetic landscape of both adaptive and innate immune cells. In the resting phase, immune cells display an inactive status, compatible with that of the closed conformation of the chromatin in the promoter regions of genes related to an active immune response (36). Upon vaccination, the APCs undergo dynamic DNA methylation-demethylation and histone acetylation-deacetylation processes (37), bringing them in an active transcriptional state, which allows efficient antigen presentation to T lymphocytes. The encounter of APCs with T cells unleashes a highly active transcriptional program that requires extensive remodeling of the epigenetic landscape of the activated T cells (38). In B cells, epigenetic regulations modulate somatic hypermutation and class switch DNA recombination during B cell activation and differentiation (39). Specific mechanisms, such as *de novo* DNA methylation, influence the differentiation of naive B cells into plasma cells. The differentiation and long-term maintenance of memory T and B cells relies on a specific DNA methylation signature that correlates with activation-induced gene expression (40).

The correct induction and maintenance of these metabolic and epigenetic modifications are crucial for the induction of an effective immune response and the generation of long-lived memory responses. These responses ensure long-term protection against the target pathogen, but they also have heterologous effects. For instance, vaccination against tetanus induces changes in the DNA methylation status of specific B cell development-related CpG islands, leading to decreased circulating IgE levels and a lower risk of asthma at 18 years of age (41). Wide DNA methylation after vaccination against influenza is associated with a humoral response to vaccination, cell differentiation signaling, and antigen presentation (42). Yellow fever vaccination induces dynamic variations in the of DNA methylation of the regulatory regions of the genome of CD8^+^ T cells, facilitating the differentiation into memory T cells or providing a signal for premature termination of antiviral functions (43). Similarly, epigenetic repression of naive-cell-associated genes in effector CD8 T cells is reversible in cells that develop into long-lived memory CD8 T cells, while effector genes remain nonmethylated (44).

## PHARMACOLOGICAL AMPLIFICATION OF THE EFFECTS OF VACCINES

**FIG 2:**
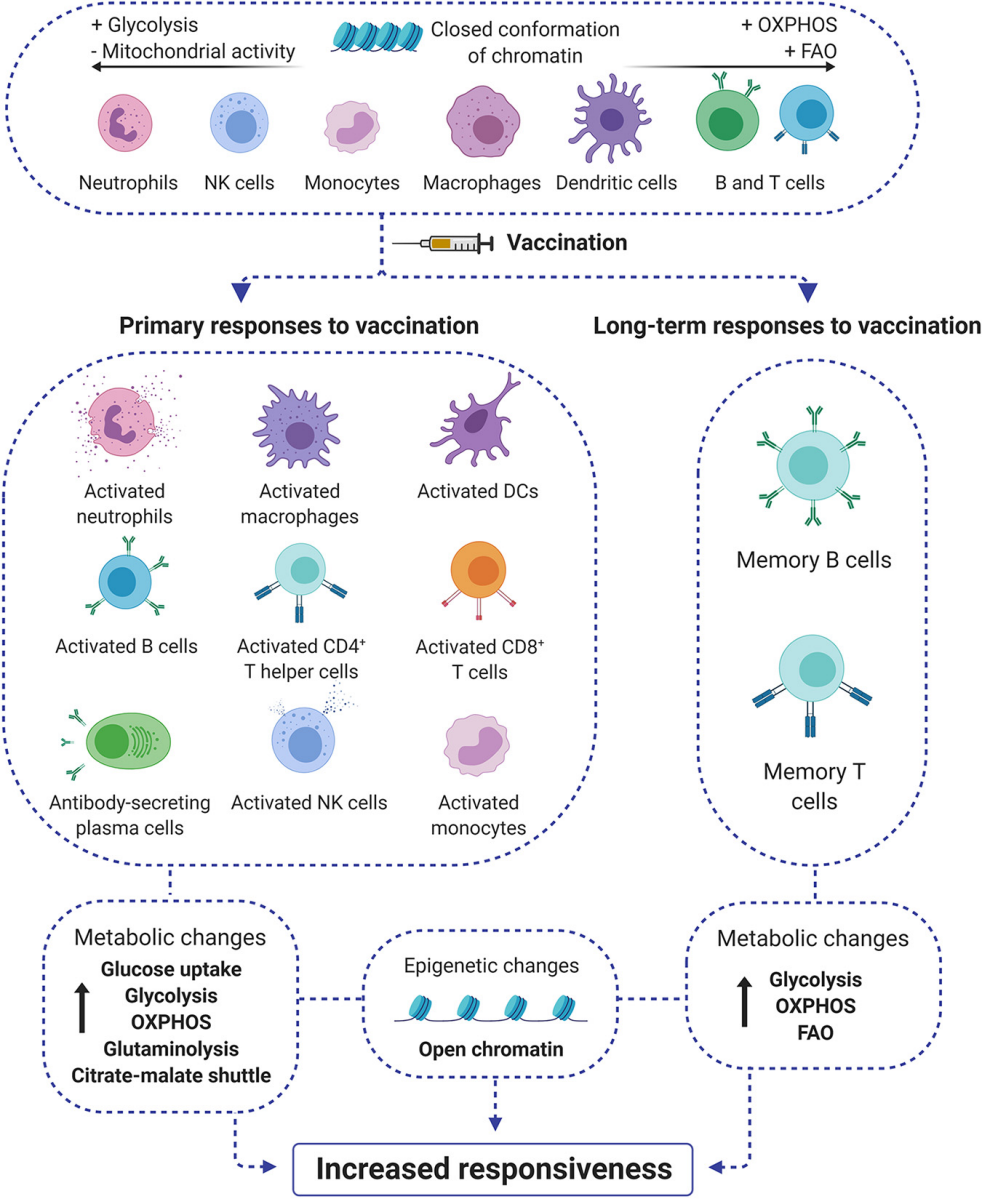
Vaccination triggers large metabolic and epigenetic rewiring in the adaptive and innate immune system. In the resting state, immune cells do not present high energetic and metabolic requirements, relying mostly on efficient processes, such as fatty acid oxidation (FAO) or oxidative phosphorylation (OXPHOS). The chromatin of cells prior to vaccination is in a closed conformation, in line with low transcriptional activity. After vaccination and subsequent activation of primary and long-term responses to vaccination, immune cells greatly increase their biosynthetic demands and use different metabolic routes and nutrients to fulfill their increased energetic and nutritional requirements. Their chromatin unfolds, allowing the transcription of inflammatory factors. The specific memory T and B cells that stay in circulation and in tissues do not go back to baseline, but they keep an increased metabolic activity and chromatin remains in the open conformation to allow their increased responsiveness when they encounter a subsequent pathogenic stimulus.

There is a growing number of pharmacological compounds that can potentiate or hinder the activities of specific metabolic pathways and epigenetic mechanisms. Many of these drugs are safe to use in humans and are being tested in clinical trials (45, 46). The use of pharmacological modulators of the metabolic and epigenetic responses has the potential to fine-tune specific and nonspecific effects of vaccines. For instance, skewing the metabolic activity of activated T cells toward fatty acid oxidation influences the activation and polarization process favoring the cytotoxic responses of vaccines (47), while the blockade of the metabolic switch from oxidative phosphorylation to aerobic glycolysis impairs the ability of these cells to produce gamma interferon (IFN-γ) (48). Similarly, the use of metformin inhibits the induction of trained immunity by BCG in monocytes, as shown by lower cytokine production and lactate production upon secondary stimulation (49), but on the other hand, it increases the immune responses after vaccination in an experimental model of anticancer vaccination (47). These are just some examples of how pharmacological modulation, interference, and fine-tuning of metabolic and epigenetic mechanisms in adaptive and innate immune cells can affect the responses to vaccines (50) and exemplify the potential of these strategies in the design and development of new vaccines and vaccination strategies (Fig. 2). We therefore propose that one important future path for the design of vaccines with improved efficacy is to add a metabolic and/or epigenetic modulator with the capacity to amplify the memory and effector function of immune cells. We propose the term “amplifier” for such vaccine additives, and we pose that such future vaccines will have three major components: antigen, adjuvant, and amplifier.

The immunomodulatory properties of a new generation of vaccines based on the modulation of metabolic and epigenetic effects would be able to cover a great range of diseases and syndromes of communicable and noncommunicable origins. Every condition that involves the participation of the immune system would be susceptible to intervention. In this regard, it would be important to distinguish between the prophylactic and the therapeutic potential of vaccines. Vaccines are often associated with prophylactic effects, priming, and training the immune system before the exposition to the pathogen, thus blocking the progression and the transmission of the infection. In this case, the desirable immune response is durable, offering protection for years and generating long-term innate and adaptive responses without the induction of a strong acute inflammatory response, since there is no need to combat an already-existing infection. On the other hand, the modulation of the effects of therapeutic vaccines should be more focused on the generation of robust acute responses to immediately activate the immune system and eliminate the threat, either a pathogen or a noncommunicable disease. The nature of these pharmacological interventions in the responses to vaccine can be divided mainly between modulation of the metabolic and the epigenetic responses to vaccines.

### Metabolic modulation.

There are several pathways that can be targeted for metabolic modulation in vaccines. They include the following.

### Glycolysis.

After activation, virtually all cell subsets undergo an increase in the uptake and the oxidation of glucose to pyruvate, which is transformed into lactate through aerobic glycolysis or employed as a precursor of acetyl coenzyme A (acetyl-CoA) for the tricarboxylic acid (TCA) cycle and oxidative phosphorylation (OXPHOS) (51). Any alteration in these pathways has a profound impact on immune functions. For instance, the loss of a mitochondrial protein (cyclophilin D) in T cells enhances glycolysis and OXPHOS and increases T cell proliferation/activation, which leads to increased pulmonary immunopathology in tuberculosis (52). Thus, the pharmacological targeting of glycolysis is a clear candidate for the modulation of immune responses to vaccines. For example, 2-deoxyglucose, an inhibitor of the enzyme that catalyzes the first step of glycolysis by inhibiting hexokinase, has been evaluated in multiple clinical trials as a means to decrease glucose and modulate immune responses (53). Another compound, sodium oxamate, acts as a lactate dehydrogenase inhibitor, disrupting the conversion of pyruvate into lactate, thereby increasing the flux of pyruvate into the TCA cycle and increasing the activity of OXPHOS (54).

### TCA cycle and OXPHOS.

Cells from both the adaptive and the innate immune system undergo an increase in their TCA cycle activity coupled with OXPHOS in the electron transport chain of mitochondria after immune activation (27, 53, 55). There are multiple compounds that may be used to modulate the activities of these pathways, such as dichloroacetate, an inhibitor of AMP-activated protein kinase (AMPK), approved as a therapy against mitochondrial disorders and pulmonary hypertension and, in clinical trials, against carcinoma (56). This compound favors the transformation of pyruvate into acetyl-CoA, fueling the TCA cycle. On the other hand, the activation of AMPK through the use of the agonist compounds AICAR (5-aminoimidazole-4-carboxamide-1-β-d-ribofuranoside) and phenoformin suppresses IFN-γ production by T effector cells (57). Metformin, a biguanide widely used against type II diabetes and in multiple clinical trials against other diseases, also modulates the activity of the TCA cycle and OXPHOS, as well as fatty acid oxidation (58). Dimethyl itaconate, an analogue of the endogenous TCA cycle metabolite itaconate, exerts powerful immunomodulatory actions via the TCA cycle in innate immune cells (59). The use of analogues of endogenous metabolites, such as succinate and fumarate, may also be very interesting, since these compounds have shown powerful immunomodulatory properties linked to their metabolic and epigenetic effects (60). In this regard, the production of succinate by macrophages induces inflammatory mechanisms through the activation of HIF-1α and the production of interleukin 1β (IL-1β) (61), while the treatment of monocytes with methyl fumarate causes metabolic and epigenetic reprogramming of these cells, leading to the induction of trained immunity (60, 62).

### Fatty acid oxidation.

The metabolic route of fatty acid oxidation is fundamental to providing energy and nutrients to resting T and B cells, which can also be modulated to amplify the effects of vaccines. In this regard, compounds like etomoxir (63), an inhibitor of fatty acid oxidation that has already been employed in several clinical trials, or C75, an inhibitor of fatty acid synthase that has shown activity in several models of cancer (64), might have great potential to modulate the effects of vaccines in the future. Metformin, already mentioned as a modulator of the TCA cycle and OXPHOS, has already been successfully employed to increase CD8^+^ T memory responses and improve the efficacy of a vaccine in a murine cancer experimental model through the activation of fatty acid oxidation (47).

### Cholesterol metabolism.

Different metabolic pathways associated with cholesterol have been shown to play a fundamental role in the activation of both innate and adaptive immune cells. Cholesterol and its derivatives play a fundamental role in the formation of the plasma membrane and in intracellular trafficking and the transcriptional regulation of the immune responses, controlling the signaling of proinflammatory macrophages or the proliferation of activated B and T cells (65). In this regard, the use of modulators of the synthesis of cholesterol, such as statins and their derivatives, widely prescribed drugs, may have the potential to impact the different types of immune responses to vaccines.

### Amino acid metabolism.

A growing number of reports are showing how amino acid metabolism plays a central role in the immune activation after microbial stimulation (66). The relationship between amino acid metabolism and immune responses is becoming more and more evident, with different works showing the crucial importance of glutamine in immune activation (60) or the role of tryptophan and arginine in autoimmune diseases (67). With this in mind, the use of pharmacological modulators of several pathways related to the metabolism of amino acids, such as BPTES {*N*,*N*′-[thiobis(2,1-ethanediyl-1,3,4-thiadiazole-5,2-diyl)]bis-benzeneacetamide}, an inhibitor of glutaminolysis, or DFMO (dl-α-difluoromethylornithine [hydrochloride hydrate]), a modulator of polyamine metabolism, both used in clinical trials with human subjects (68, 69), could be employed to fine-tune the immune responses to vaccines.

### Epigenetic modulation of the responses to vaccines. (i) Histone modification.

The deposition of acetylation and methylation marks in different regions of the histones is one of the main regulatory mechanisms of immune responses that impact immunity in both the short and the long term. This dynamic interplay depends on the activities of several key enzymes that control the addition or removal of the acetyl or methyl groups that affect the accessibility of the transcription factors to the promoters or regulatory regions of the genes involved in immune responses. These enzymes are histone acetylases (HAT), histone deacetylases (HDAC), histone methyltransferases (HMT), histone lysine demethylases (KDM), or bromodomain and extraterminal motif (BET) proteins (70). Due to the central role of histones in the regulation of the epigenetic landscape of cells, there is a growing interest in developing a number of pharmacological modulators targeting histones. For example, BET inhibitors (I-BETs) are a class of drugs that bind the bromodomains of BET proteins, which recognize and read acetylated lysines of histone and transcription factors (71) and prevent protein-protein interaction between BET proteins and their targets. These I-BETs present strong activities as modulators of immune responses and have shown great efficacy as modulators of antitumor responses; they may be excellent candidates for use as amplifiers of the responses to vaccines in the future (72, 73). Multiple ligands and inhibitors of HDAC, HAT, HMT, and KDM that are either under development or being tested in clinical trials for neuropsychiatric disorders, cancer, and autoimmune diseases (74) may be employed to modulate the accessibility of the promoter regions for proinflammatory genes after vaccination. Among these enzymes, the modulation of lysine methyltransferase G9a has shown a promising potential in T helper cells to amplify their responses to vaccination (75).

### (ii) DNA methylation.

Through this process, methyl groups can be added or removed from cytosine groups at the DNA of cells, altering transcription. Typically, the addition of a methyl group to a gene promoter region represses its transcription, while the removal of the methylation mark facilitates transcription. The processes involved in DNA methylation are controlled by a group of enzymes called DNA methyltransferases (DNMT). Removal of DNA marks from DNA requires other enzymes, such as the 10 to 11 translocation (TET) proteins, activation-induced cytidine deaminase (AID), and thymine DNA glycosylase (TDG), which also play a fundamental role in this process (76). Since the pathogenesis of diseases such as cancer often involves alterations in the methylation patterns of suppressor genes (77), there is an interest in the rapid development of pharmacological modulators of these enzymes. Therefore, a large number of small molecules, such as decitabine, tazemetostat, and molecules based on 8-hydroxyquinoline, among many others (78), or peptides, such as menin (79), have been developed in recent years, and some have already been included in clinical trials. The large number of molecules that can modulate DNA methylation thus offers the potential for modulation of DNA methylation-related processes in immune cells after vaccination, and this should be taken into account in the development of a new generation of vaccines.

### (iii) miRNAs.

MicroRNAs (miRNAs) are small molecules of RNA (21 to 25 nucleotides) that regulate gene expression at the posttranscriptional level. They usually act on gene expression by silencing or degrading mRNAs. miRNAs play a major role in many aspects of immunity and vaccination (80). Efforts are focused on developing vaccines based on the use of miRNAs to attenuate viral replication, with promising results. Moreover, the human hosts can also regulate the levels of endogenous miRNAs after vaccination. In this sense, Drury et al. studied the expression of miRNAs in the sera of children 21 days after vaccination with a pandemic influenza (H1N1) vaccine, finding 19 miRNAs that were differentially expressed, even though their findings could not be fully validated (81). miRNAs can be considered epigenetic regulators which affect the protein levels of targeted mRNAs without modifying the gene sequences. As miRNAs are also susceptible to regulation by epigenetic mechanisms, they can have a dual role in the development of a next generation of vaccines; both miRNAs administered in vaccines and endogenous miRNAs could be the target of pharmacological modulators administered through vaccination.

## FIELDS FOR APPLICATION OF A NOVEL GENERATION OF VACCINES

### The rising problem of antimicrobial resistance.

Globally, the morbidity and mortality associated with antimicrobial resistance (AMR) due to bacterial and fungal infections, such as methicillin-resistant Staphylococcus aureus, Streptococcus pneumoniae, or Candida auris, among many others, is increasing at an alarming rate (Table 1). Therefore, the WHO has developed a global strategy for the containment of AMR and urged governments and health agencies to support the search of new strategies against AMR (82). One of the most important approaches to prevent and combat infections is vaccination. Despite their effectiveness, there is little attention for the potential of vaccines to reduce mortality and morbidity (83).

**TABLE 1:** WHO priority pathogen list for research and development of new antibiotics

Priority	Organism(s)	Comment(s)
1 (critical)	Acinetobacter baumannii	Carbapenem resistant
	Pseudomonas aeruginosa	Carbapenem resistant
	*Enterobacteriaceae*	Carbapenem resistant, extended-spectrum beta-lactamases

2 (high)	Enterococcus faecium	Vancomycin resistant
	Staphylococcus aureus	Methicillin resistant, vancomycin intermediate and resistant
	Helicobacter pylori	Clarithromycin resistant
	*Salmonellae*	Fluoroquinolone resistant
	Neisseria gonorrhoeae	Cephalosporin resistant, fluoroquinolone resistant
	*Campylobacter* spp.	Fluoroquinolone resistant

3 (medium)	Streptococcus pneumoniae	Penicillin nonsusceptible
	Haemophilus influenzae	Ampicillin resistant
	*Shigella* spp.	Fluoroquinolone resistant

There are several mechanisms by which vaccines can reduce the emergence of AMR. First, preventing bacterial infections reduces the use of antibiotics to treat such infections, therefore limiting the use of antibiotics and decreasing the generation of resistance. Second, the use of vaccines against AMR reduces the inadequate use of antibiotics in infections caused by different pathogens that are often not sensitive to the therapies prescribed. The use of vaccines also decreases the number of healthy individuals colonized by drug-resistant pathogens, a possible source for further transmission. Third, vaccines decrease the number of circulating strains that are resistant to antibiotics. For example, the use of the antipneumococcal vaccine has also reduced the incidence of antibiotic-resistant pneumococcal infections, while the introduction of the vaccine against Haemophilus influenzae type b (Hib vaccine) has virtually eliminated infections by ampicillin-resistant strains (83). Besides this, Hib vaccines have shown high efficacy in preventing invasive disease in children and have contributed to a decreased use of antibiotics and, therefore, the development of lower resistance to β-lactamases (84, 85). Similarly, vaccination with pneumococcal conjugates causes a strong decrease in inflammatory pneumococcal diseases only 7 years after their introduction (86, 87), reducing antibiotic use and the prevalence of resistant strains, which decreased in parallel (88). For its part, vaccination against influenza also decreases the incidence of secondary infections, such as pneumonia and otitis media.

Finally, live vaccines, such as BCG, yellow fever, or measles vaccines, reduce the overall infection and mortality rates in children and adults (18, 89). Importantly, different studies have shown that the protective effects of vaccines last at least for several months but may be modified or even reversed when a non-live vaccine is given (90, 91), so the design of correct vaccination and immunization schemes is crucial to grant maximum protection. Vaccination with BCG increases the cytokine production and responsiveness of monocytes, providing protection against heterologous diseases such as yellow fever (92) and malaria (93). Despite these successes, it is fundamental for us to develop new vaccines against antibiotic-resistant bacteria. With all this in mind, a new generation of vaccines may help reduce the incidence of viral infections and secondary bacterial infections, which usually require antimicrobial treatment.

### Emerging infections and old pathogens.

The amplification of the effects of vaccines offers great potential against emerging infections or pathogens for which we do not yet have an effective vaccine. Eliciting the activities of the immune system in a nonspecific manner can be used to decrease the rates of infection and transmission of diseases for which a vaccine is not available. Even in cases such as the COVID-19 pandemic, in which unprecedented global efforts have been invested in the quick development of a specific effective vaccine, it is not possible to develop, test, manufacture, and distribute a vaccine in less than at least 1 or 2 years. The development of a novel generation of “bridge vaccines” with amplified, nonspecific effects against different types of pathogens can be used to decrease the morbidity and mortality of those agents for which a fully effective vaccine does not exist and protect vulnerable populations (24). Therefore, the administration of vaccines with elicited nonspecific effects can bridge the period of time necessary to develop specific vaccines and treatments and decrease the rates of infection and transmission, reducing the global burden. Likewise, the immunomodulatory effects of these vaccines may be used as prophylaxis and adjuvant treatments to modulate and amplify immune responses against well-known diseases without a fully effective vaccine, such as those for tuberculosis, HIV, or malaria.

### Populations at risk: the elderly and immunocompromised patients.

An increasing body of evidence, both at the epidemiological and the immunological level, argue for a sustained impairment of host defense mechanisms with age, a process termed “immunesenescence in aging” (94). This is characterized by higher susceptibility to infections and increased incidence of neoplastic disorders. The adaptive arm of the immune response suffers critical changes with age, such as lower numbers and functions of naive T cells, impaired memory responses, thymic involution, T cell repertoire skewing, and a lesser capacity to release Th1/Th17 cytokines (95). In addition, researchers have started to identify the molecular mechanisms responsible for this decline, including (but not limited to) DNA damage, proteotoxic stress, defects in gene transcription, and dysregulation of the ubiquitin proteasome pathway (96). The decreased effectiveness of the immune system with old age is now considered a marker of health and a predictor of longevity (97). Vaccination in the elderly (e.g., against influenza and pneumococcus) is seen as a crucial tool to decrease the morbidity and mortality due to these severe infections (98), but the defects of adaptive immune responses lead to a diminished effectiveness of vaccination in the elderly. We therefore need to improve our understanding of the underlying mechanisms of immune senescence and examine how we can improve vaccination responses in elderly people. A recent study showed that prophylactic vaccination of elderly individuals with BCG decreased the incidence of respiratory tract infections without differences in the frequencies of adverse events (24), showing the potential of trained immunity to boost immune responses in the elderly population.

The WHO Strategic Advisory Group of Experts on Immunization recently concluded that evidence suggests a beneficial effect of immunization with the BCG and live attenuated measles vaccine on all-cause mortality in high-risk populations (18). Interestingly, recent studies from the Human Functional Genomics Project have shown that, in contrast to adaptive immunity responses, innate immune responses seem to be intact in elderly individuals (99). This opens the exciting possibility that, in addition to the use of metabolic or epigenetic amplifiers that increase the responsiveness of the innate immune system, innate immune responses may represent a novel approach for vaccination in the elderly. Therefore, the pharmacological modulation that plays between the host (epi)genome, the metabolic programs of myeloid cells in the microbiome, and environmental factors can lead to the improvement of vaccine efficacy in this population.

Beyond the elderly, the number of immunocompromised patients has increased in recent years as a result of treatments provided for malignancies and autoimmune disorders (100). Immunization of these patients is essential since they are at greater risk of infections while being treated for other diseases, like cancer. The administration of vaccines to these individuals can be used as a strategy to boost the activity of their immune system and decrease their morbidity and mortality due to infections. However, due to the immunocompromised state of these patients, they cannot receive live vaccines. In this sense, the addition of an amplifier of the responses to non-live vaccines could be employed to increase the protection offered by these vaccines and take advantage of the epigenetic and metabolic reprogramming of the cells to provide protection against disease.

## CONCLUSIONS AND FUTURE CHALLENGES

**FIG 3:**
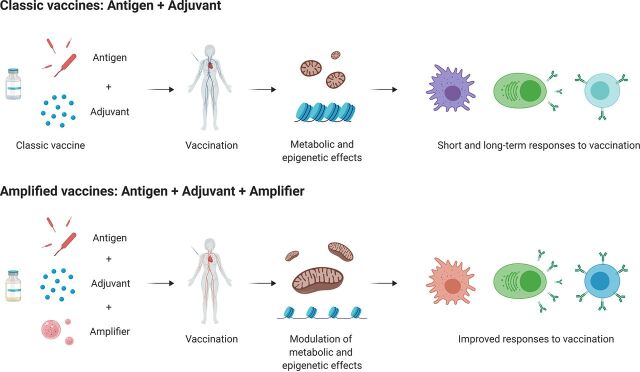
Addition of epigenetic and/or metabolic modulators to vaccines as a new approach to increase efficacy. While the combination of an antigen and an adjuvant has been proven useful to develop the current generation of vaccines, pharmacological modulation of the metabolic and epigenetic mechanisms involved in the adaptive and innate immune cells after vaccination can potentiate, fine-tune, and improve responses to vaccines; such modulation can also use these potential mechanisms in the design and development of new vaccines and vaccination strategies.

Although vaccines are very effective tools to reduce the incidence of infectious diseases and the use of antibiotics, they are still a long way from their full potential. In addition, there are still many important infectious diseases for which no effective vaccine is available (e.g., TB, malaria, HIV), as well as groups at risk with poor responses to the current vaccines (e.g., elderly individuals). We should therefore consider amplification and fine-tuning of the effects of vaccines through the use of metabolic and epigenetic modulators, which we term vaccine amplifiers (Fig. 3). Such approaches not only improve the efficacy of current vaccines but also may eventually potentiate the responses triggered by vaccines under conditions that occur with low activity of the immune system. These conditions include patients with immune deficiencies, sepsis-induced immunoparalysis, people at high risk of developing infectious diseases, patients with certain types of malignancies (including those patients undergoing treatment with immunotherapy), or individuals affected by drug-resistant pathogens. Besides this, the addition of an amplifier to vaccines not only can enhance host immunity to infections but also target the metabolic and epigenetic factors of pathogens which are evolutionary conserved, facilitating the clearance of new pathogens (101).

Thus, we hope that in the coming years, more efforts will be focused on defining the metabolic and epigenetic pathways in the immune cells for developing novel pharmacological modulators targeting these pathways. We envision that increased efficacy of vaccines through amplifiers will be a novel approach to combat human diseases in subsequent years.
